# ESCRT-0 dysfunction compromises autophagic degradation of protein aggregates and facilitates ER stress-mediated neurodegeneration via apoptotic and necroptotic pathways

**DOI:** 10.1038/srep24997

**Published:** 2016-04-26

**Authors:** Ryuji Oshima, Takafumi Hasegawa, Keiichi Tamai, Naoto Sugeno, Shun Yoshida, Junpei Kobayashi, Akio Kikuchi, Toru Baba, Akira Futatsugi, Ikuro Sato, Kennichi Satoh, Atsushi Takeda, Masashi Aoki, Nobuyuki Tanaka

**Affiliations:** 1 Division of Neurology, Department of Neuroscience and Sensory Organs, Tohoku University Graduate School of Medicine, Sendai 980-8574, Japan; 2 Division of Cancer Biology and Therapeutics, Miyagi Cancer Center Research Institute, Natori 981-1293, Japan; 3 Division of Pathology, Miyagi Cancer Center Research Institute, Natori 981-1293, Japan; 4 Division of Cancer Stem Cell, Miyagi Cancer Center Research Institute, Natori 981-1293, Japan; 5 Department of Basic Medical Science, Kobe City College of Nursing, Hyogo 651-2103, Japan; 6 Department of Neurology, Sendai-Nishitaga Hospital, Sendai 982-8555, Japan

## Abstract

Endosomal sorting required for transport (ESCRT) complexes orchestrate endo-lysosomal sorting of ubiquitinated proteins, multivesicular body formation and autophagic degradation. Defects in the ESCRT pathway have been implicated in many neurodegenerative diseases, but the underlying molecular mechanisms that link them to neurodegeneration remain unknown. In this study, we showed that forebrain-specific ablation of ESCRT-0/Hrs induced marked hippocampal neuronal cell loss accompanied by the accumulation of ubiquitinated proteins, including α-synuclein, TDP-43 and huntingtin as well as the autophagic substrate SQSTM1/p62. Consistent with this, silencing of Hrs in cultured cells not only led to α-synuclein and TDP-43 accumulation in addition to impaired autophagic flux but also suppressed cell viability through the induction of ER stress followed by the activation of JNK and RIPK1, a key regulator of necroptosis. Moreover, necrostatin-1, a specific inhibitor of RIPK1, and pan-caspase inhibitors partially reduced the neurotoxicity in the Hrs-silenced cells. Altogether, these findings suggest that the disruption of ESCRT-0/Hrs in the nervous system compromises autophagic/lysosomal degradation of neurodegenerative disease-related proteins, which thereby triggers ER stress-mediated apoptotic and necroptotic cell death.

Selective neuronal loss accompanied by specific protein aggregation is the histopathological hallmark of neurodegenerative diseases. Although the abnormal proteins responsible for each disease are different in structure and function, all neurodegenerative disorders share the common process of protein misfolding and aggregation^1^. These aggregates directly and indirectly attack cellular components, leading to neuronal cell death^2^. In order to fight against these continuous threats, cells have evolved ingenious defense mechanisms that act either to facilitate refolding of misfolded proteins by molecular chaperones or to remove them by proteolytic degradation machinery, including the ubiquitin-proteasome system (UPS) and autophagy-lysosome pathway (ALP)^3^.

Endosomal sorting complex required for transport (ESCRT) proteins form multimolecular complexes that control multivesicular body (MVB) formation and transport ubiquitinated cargo proteins^4^. This evolutionarily conserved machinery consists of three distinct but cooperative functions: first, hepatocyte growth factor-regulated tyrosine kinase substrate (Hrs), a vital element of ESCRT-0, recognizes ubiquitinated cargoes; second, ESCRT-0 recruits ESCRT-I and II, promoting perimeter membrane deformation in concert with ESCRT-III, which facilitates sorting of the cargo into endosomal invaginations; third, vacuolar protein sorting 4 (VPS4) catalyzes the final membrane abscission to form MVB^5^. MVB then fuses with the lysosome, where the intraluminal vesicles and cargoes are degraded. Alternatively, MVB participates in (macro)autophagic proteolysis, where it fuses with the autophagosome to generate the amphisome, a ‘prelysosomal hybrid’ organelle^6^.

Growing evidence has shown that several neurodegenerative diseases, including Alzheimer’s disease (AD), Parkinson’s disease (PD), and amyotrophic lateral sclerosis (ALS), are associated with defects in the endo-lysosomal pathway^7^,^8^,^9^,^10^. Of them, ESCRT has attracted special attention since abnormalities in ESCRT complexes have been discovered in a wide variety of neurodegenerative diseases. For example, mutations in the charged multivesicular body protein 2B (CHMP2B), an ESCRT-III subunit, were identified in familial cases of frontotemporal dementia (FTD) and ALS^11^. Furthermore, mutations in STAMBP/AMSH (signal transducing adaptor molecule binding protein/association molecule with STAM SH3 domain) cause microcephaly-capillary malformation syndrome^12^. Moreover, immunoreactivity against CHMP2B and/or VPS4 has been found in neuronal inclusions in AD and PD^13^,^14^,^15^. The importance of ESCRT machinery in neurodegenerative process is also supported by recent studies showing that the depletion of ESCRT-0/Hrs inhibits the targeting of amyloid precursor protein to the MVB and lysosome, which eventually leads to increased intracellular Aβ accumulation^16^,^17^. These findings indicate that ESCRT is closely involved in the pathogenic processes that lead to neurodegenerative diseases^13^,^14^. However, the downstream events that connect ESCRT dysfunction and neuronal cell death remain little understood.

In the current study, we find that ESCRT-0/Hrs is indispensable for the autophagic clearance of neurodegeneration-related proteins such as α-synuclein (αS), TAR DNA-binding protein (TDP-43) and huntingtin and for the survival of hippocampal neurons in mammals. In particular, this work provides the novel insight that the loss of Hrs results in insufficient autophagic clearance and enhanced ER stress, thereby triggering c-Jun N-terminal kinase (JNK) activation and subsequent apoptotic and necroptotic neuronal cell death.

## Results

### Forebrain-specific Hrs knockout mice show lethal neurological phenotypes

**Figure 1:**
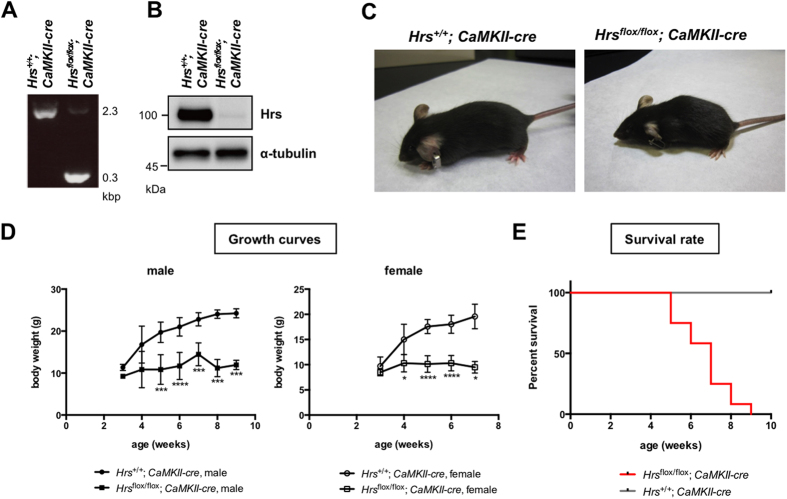
Forebrain-specific deletion of ESCRT-0/Hrs shows lethal neurological phenotype in mice. (**A**) Genomic PCR analysis of the *Hrs*^+/+^*; CaMKIIα-cre* and *Hrs*^*flox/flox*^*; CaMKIIα-cre* mice. The PCR products included a 0.3 kbp band for the Hrs^Δ2–4^ allele and a 2.3 kbp band for the wild-type allele. (**B**) Immunoblot analysis of Hrs in the mouse forebrain. Lysates from *Hrs*^+/+^*; CaMKIIα-cre* and *Hrs*^*flox/flox*^*; CaMKIIα-cre* forebrains were separated by SDS-PAGE and blotted with an anti-Hrs antibody. (**C**) The appearance of *Hrs*^+/+^*; CaMKIIα-cre* and *Hrs*^*flox/flox*^*; CaMKIIα-cre* mice. *Hrs*^*flox/flox*^*; CaMKIIα-cre* mice were smaller in size at 4 weeks of age. (**D**) Growth curves for *Hrs*^+/+^*; CaMKIIα-cre* and *Hrs*^*flox/flox*^*; CaMKIIα-cre* mice. Significant growth retardation was observed in the *Hrs*^*flox/flox*^*; CaMKIIα-cre* mice compared with the *Hrs*^+/+^*; CaMKIIα-cre* mice. Values are expressed as means ± standard errors. *p < 0.05, ***p < 0.005, ****p < 0.001 (unpaired Student’s *t*-test; male, n = 9; female, n = 8). (**E**) Survival rate of *Hrs*^+/+^*; CaMKIIα-cre* and *Hrs*^*flox/flox*^*; CaMKIIα-cre* mice. All the *Hrs*^*flox/flox*^*; CaMKIIα-cre* mice died by 9 weeks of age.

As an initial step to investigate the functional significance of Hrs in the brain *in vivo*, we generated *Hrs* conditional knockout mice by crossing *CaMKIIα (Calcium/ calmodulin-dependent protein kinase II α)*-*cre* mice with *Hrs-floxed* mice. The *Hrs*^*flox/flox*^; *CaMKIIα-cre* mice were viable and producing offspring in a Mendelian ratio (Fig. 1A). Immunoblot analysis showed that the Hrs protein was almost completely absent in the brain of *Hrs*^*flox/flox*^; *CaMKIIα-cre* mice (Fig. 1B). Interestingly, the *Hrs*^*flox/flox*^; *CaMKIIα-cre* mice were smaller in size at 4 weeks of age, and thereafter, significant growth retardation and feeding difficulties were observed in the conditional knockout mice compared with the *Hrs*^+/+^; *CaMKIIα-cre* mice (Fig. 1C,D). By 9 weeks of age, all the *Hrs*^*flox/flox*^; *CaMKIIα-cre* mice had died, whereas none of the control mice had died, indicating that conditional knockout of *Hrs* in the forebrain resulted in a lethal phenotype (Fig. 1E).

### Hippocampal neuronal loss and protein aggregate accumulation in Hrs knockout mice

**Figure 2:**
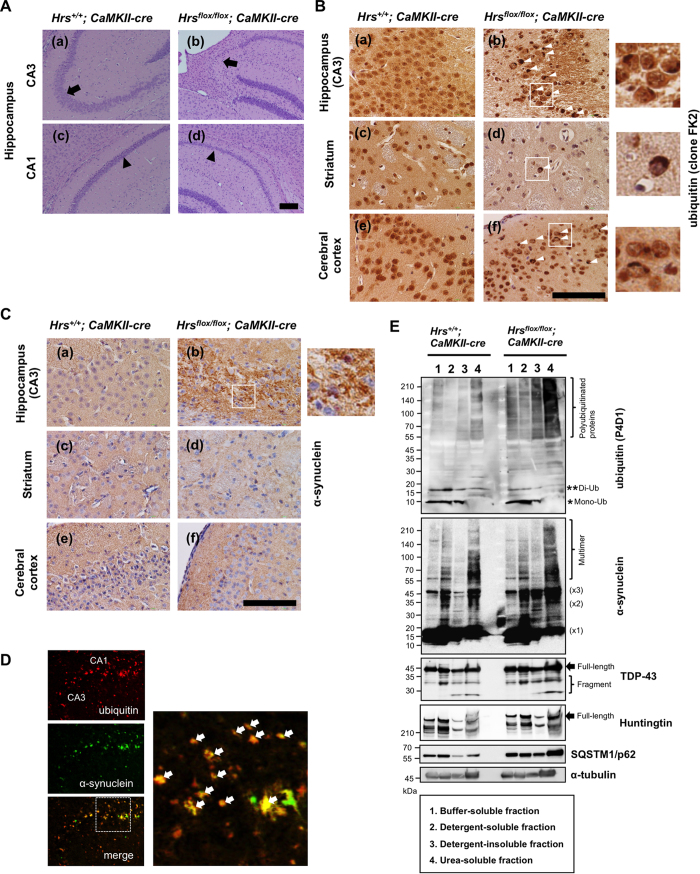
Hippocampal neuronal loss and protein aggregate accumulation in Hrs knockout mice. (**A**) Neuronal cell loss in the hippocampus of the *Hrs*^*flox/flox*^*; CaMKIIα-cre mice* at 7weeks of age. The brains of the *Hrs*^*flox/flox*^*; CaMKIIα-cre* mice exhibited marked neuronal cell loss in specific regions of the hippocampus: CA3 (a, b; *black arrow*) and CA1 (c, d; *black arrowhead*). Scale bar, 1000 μm. (**B**,**C**) Immunohistochemistry of hippocampal area CA3 (a, b), striatum (c, d), and cerebral cortex (e, f) sections from *Hrs*^+/+^*; CaMKIIα-cre* (a, c, e) and *Hrs*^*flox/flox*^*; CaMKIIα-cre* (b, d, f) mice at 7 weeks of age (Fig. 2B). Accumulation of ubiquitin-positive inclusions (*white arrowhead* in Fig. 2B) and α-synuclein-positive inclusions (b in Fig. 2C) in the *Hrs*^*flox/flox*^*; CaMKIIα-cre* brain. The white square indicates the magnified area. Scale bar, 100 μm. (**D**) ubiquitin (red) and α-synuclein (green) positive inclusions (*white arrow*) were observed close together in hippocampal areas CA1 and CA3 of the *Hrs*^*flox/flox*^*; CaMKIIα-cre* brain by immunofluorescence analysis. The white square indicates the magnified area. (**E**) Protein extraction of the buffer-soluble, detergent-soluble, detergent-insoluble, and urea-soluble fractions from the mouse brains. Each lysate was separated by SDS-PAGE and blotted with anti-ubiquitin, anti-α-synuclein, anti-TDP-43, anti-huntingtin, and anti-p62 antibodies.

In the 2- and 3-week-old brains, no histopathological differences were observed between the *Hrs*^*flox/flox*^; *CaMKIIα-cre* and *Hrs*^+/+^; *CaMKIIα-cre* mice (data not shown). On the other hand, the brains of the *Hrs*^*flox/flox*^; *CaMKIIα-cre* mice at 7 weeks of age exhibited marked neuronal cell loss in specific regions of the hippocampus (Fig. 2A, a–d). In the conditional knockout mice, area CA3 was the most severely affected, and almost all of the pyramidal cell layer was lost. Likewise, the pyramidal cell layer of area CA1 in the conditional knockout mice was thinner than that in the control mice. To determine whether the lesions were associated with abnormal protein accumulation, we next examined immunoreactivity for neurodegenerative disease-related proteins. Neuronal cells in hippocampal area CA3, the striatum and the cerebral cortex exhibited a basal amount of ubiquitin in the nucleus (Fig. 2B, a,c,e). Surprisingly, dense accumulation of ubiquitinated proteins was observed within the cytoplasm of degenerating neurons in the three regions described above in the *Hrs*^*flox/flox*^; *CaMKIIα-cre* mice at 7 weeks of age (Fig. 2B, b,d,f, *arrowheads*). Note that the accumulation of ubiquitinated proteins was prominent in hippocampal area CA3 compared to other regions including cerebral cortex and striatum. Furthermore, the accumulation of ubiquitinated proteins had already observed in the brain of *Hrs*^*flox/flox*^*; CaMKIIα-cre* mice by 5 weeks at which the neuronal cell loss has not yet started (data not shown). Another intriguing finding was that numerous small aggregates of αS, a major constituent of Lewy body in PD, appeared in the CA3 hippocampal neurons of Hrs knockout mice (Fig. 2C), and immunofluorescence images showed that they indeed colocalized with each other (Fig. 2D). We next examined the solubility of accumulated proteins in the mouse forebrain (Fig. 2E). The amounts of monomeric and dimeric free ubiquitin in the buffer- and detergent-soluble fractions remaining almost unchanged irrespective of the expression of Hrs; however, there was a conspicuous increase of polyubiquitinated proteins in the insoluble fraction in the brains of the Hrs knockout mice. In agreement with this, dimeric, trimeric and multimeric forms of αS species were increased in the urea-soluble fraction of the *Hrs*^*flox/flox*^; *CaMKIIα-cre* mice. Likewise, TDP-43, a cardinal protein in FTD and ALS, and huntingtin, a protein responsible for Huntington’s disease, were elevated in the urea-soluble fraction of the extracts from the *Hrs*^*flox/flox*^; *CaMKIIα-cre* mice. In accordance with these findings, in the extracts from the brains of the *Hrs*^*flox/flox*^; *CaMKIIα-cre* mice, the expression level of SQSTM1/p62 was markedly augmented mainly in the detergent-insoluble and urea-soluble fractions.

### Silencing of Hrs impairs the late stage of autophagic flux

**Figure 3:**
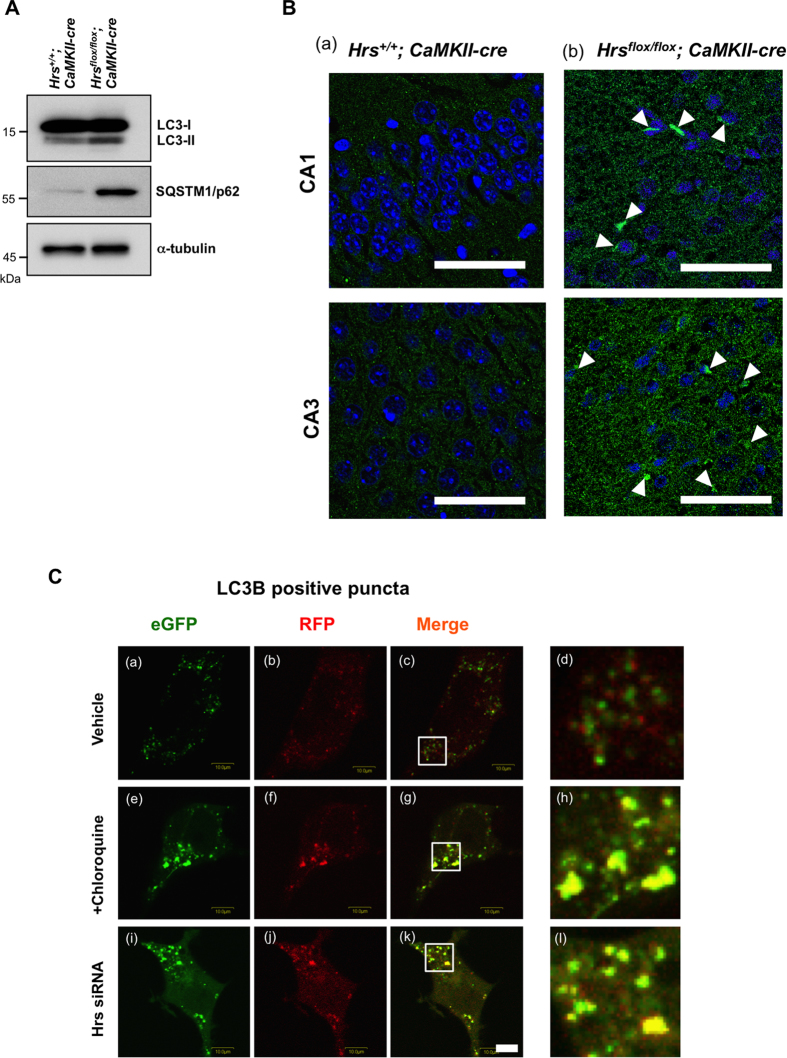
Silencing of Hrs impairs the late stage of autophagic flux. (**A**) Expression of LC3 and p62 in *Hrs^+/+^; CaMKIIα-cre* and *Hrs*^*flox/flox*^*; CaMKIIα-cre* mouse brains. (**B**) p62 immunofluorescence in hippocampal section from *Hrs*^+/+^*; CaMKIIα-cre* (a) and *Hrs*^*flox/flox*^*; CaMKIIα-cre* (b) mice. Note that *Hrs*^*flox/flox*^*; CaMKIIα-cre* mice showed the accumulation of p62-positive inclusions (*white arrowhead*) in area CA1 and CA3 of the hippocampus. Nuclei were counterstained with DRAQ7. Scale bar, 100 μm. (**C**) Monitoring autophagy flux using Hrs-silenced PC12 cells that were transfected with the RFP-GFP-LC3B construct. Cells that were transfected with scrambled siRNA were treated with vehicle (a–d) and chloroquine (e–h) as controls. The transition from the autophagosome (yellow) to the autolysosome (red) can be visualized by the specific loss of GFP fluorescence upon acidification of the autophagosome following lysosomal fusion. The white square indicates the territory magnified in Fig. 3C, d, h, and i. Scale bar, 10 μm.

The intraneuronal accumulation of ubiquitinated protein aggregates as well as the autophagic substrate p62 in the *Hrs*^*flox/flox*^; *CaMKIIα-cre* mice raised the possibility that autophagic protein degradation could be affected by the absence of Hrs. To confirm this, we checked the expression levels of the autophagosome marker LC3-II and p62 in the forebrain of the mice. Figure 3A shows a clear increase in LC3-II in the forebrain of the *Hrs*^*flox/flox*^; *CaMKIIα-cre* mice compared to the control mice. This finding was corroborated by the increase of p62-positive inclusions in hippocampal area CA1 and CA3 in the *Hrs*^*flox/flox*^; *CaMKIIα-cre* mice (Fig. 3B, *arrowheads*). To further monitor autophagy flux in the Hrs-deficient cells, we used PC12 cells that were transduced with the chimeric construct RFP-GFP-LC3B. In contrast to RFP, GFP is denatured under acidic conditions. Thus, yellow signals within the cells are interpreted as autophagosomes that have not fused with lysosomes, and the transition from autophagosome to amphisome or autolysosome can be monitored based on the selective quenching of GFP fluorescence. In the vehicle-treated cells, yellow and red punctates were observed, showing the basal levels of autophagosome and autophagolysosome formation (Fig. 3C, a–d). As expected, in the chloroquine-treated cells, many enlarged yellowish structures with a few red dots appeared, indicating inhibited fusion between the autophagosome and the acidic compartment (Fig. 3C, e–h). Similar to this, the predominant appearance of enlarged autophagosomes was observed in the Hrs-silenced cells (Fig. 3C, i–l). This outcome indicates that Hrs silencing in neuronal cells impairs autophagosome fusion with late endosomes and lysosomes.

### Silencing of Hrs induces JNK signaling activation and leads to apoptotic and necrotic cell death

**Figure 4:**
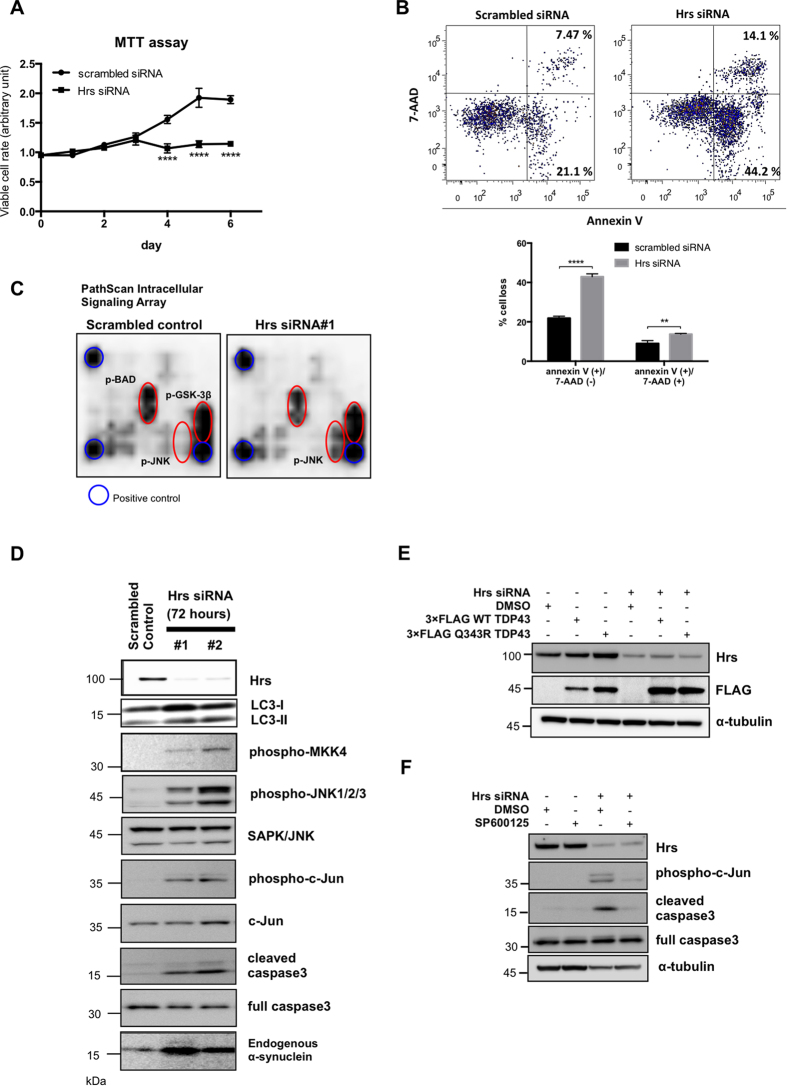
Silencing of Hrs causes the accumulation of neurodegeneration-related protein, JNK signaling activation, and leads to apoptosis and necrosis in PC12 cells. (**A**) MTT cell viability assay of Hrs-silenced PC12 cells. Values are expressed as means ± standard errors. ****p < 0.001 (unpaired Student’s *t*-test; n = 4) (**B**) Flow cytometric measurements of apoptosis and necrosis in Hrs-silenced PC12 cells. Cell death was determined by Annexin V/7-AAD double staining and analyzed as described in the Materials and Methods. The lower panel shows the percentage of Annexin V (+)/7-AAD (−) cells and Annexin V (+)/ 7-AAD (+) cells. Values are expressed as means ± standard errors. **p < 0.01, ***p < 0.005 (unpaired Student’s *t*-test; n = 3). (**C**) Results of the PathScan intracellular signaling array using lysates from control and Hrs-silenced PC12 cells. The red circles indicate positive signals for phospho-JNK, phospho-GSK-3β and phospho-BAD, whereas the blue circles indicate the positive control signal. (**D**) Western blot analysis of JNK signaling molecules in Hrs-depleted PC12 cells. Note that the loss of Hrs not only increased the expression of LC3-II and α-synuclein but also augmented MKK4, JNK and c-Jun phosphorylation as well as increased the active form of caspase-3. (**E**) Western blot analysis of TDP-43 in Hrs-delpeted PC12 cells over-expressing 3 × FLAG-tagged wt or Q343R TDP-43. Increased expression of wt and Q343R TDP-43 was observed in Hrs-silenced cells. (**F**) Phosphorylation of c-Jun and caspase-3 cleavage in Hrs-depleted cells were markedly suppressed in the presence of a JNK specific inhibitor, SP600125.

In the second set of experiments, we aimed to characterize the mode of neuronal cell death caused by the ablation of Hrs. For this purpose, we first compared the proliferation profile between control and Hrs-silenced PC12 cells using the 3-(4,5-dimethylthiazol-2-yl)-2,5-diphenyltetrazolium bromide (MTT) assay. The viability of the control cells continued to rise and reached a level that was approximate 2-fold greater than baseline at day 5. On the other hand, the viability of the Hrs-silenced cells began to gradually decrease at day 3 and returned to the initial level by day 6 (Fig. 4A). Then we performed flow cytometry analysis to distinguish apoptotic and necrotic cells. Of note, Hrs-silencing induced the Annexin V(+)/7-AAD(−) and Annexin V(+)/7-AAD(+) cell population even in the early period, indicating that the reduction in viability induced by Hrs-depletion is related to both apoptotic and necrotic cell death (Fig. 4B). In order to further unveil the molecular processes responsible for Hrs-silencing-mediated neurotoxicity, Hrs-depleted PC12 cells were subjected to the PathScan intracellular signaling array, in which phosphorylation of 18 well-characterized signaling molecules can be monitored by sandwich ELISA immunoassay (Fig. 4C). Τhe basal phosphorylation level of glycogen synthase kinase (GSK) 3β and Bcl-2-associated death promoter (BAD) remained almost unchanged in the presence or absence of Hrs; however, the phosphorylation of JNK was substantially increased in the Hrs-silenced PC12 cells. Based on this finding, we then investigated the JNK signaling cascades and caspase-3 cleavage. Western blot showed that transfection of PC12 cells with Hrs-specific siRNA not only increased the expression of LC3-II, αS, wild-type (wt) and Q343R mutant TDP-43 but also augmented JNK and c-Jun phosphorylation concomitant with MAPK kinase mitogen-activated protein kinase kinase 4 (MKK4) activation and caspase-3 cleavage (Fig. 4D,E). Note that a JNK inhibitor SP600125 substantially relieved the levels of phospho-c-Jun and caspase-3 cleavage in the Hrs-depleted cells (Fig. 4F). Together, these findings indicated that MKK4-JNK-c-Jun mediated apoptotic signaling underlies Hrs-silencing-induced neuronal cell death.

### Neurotoxicity caused by Hrs depletion depends on ER stress and subsequent JNK-mediated apoptosis and necroptosis in PC12 cells

**Figure 5:**
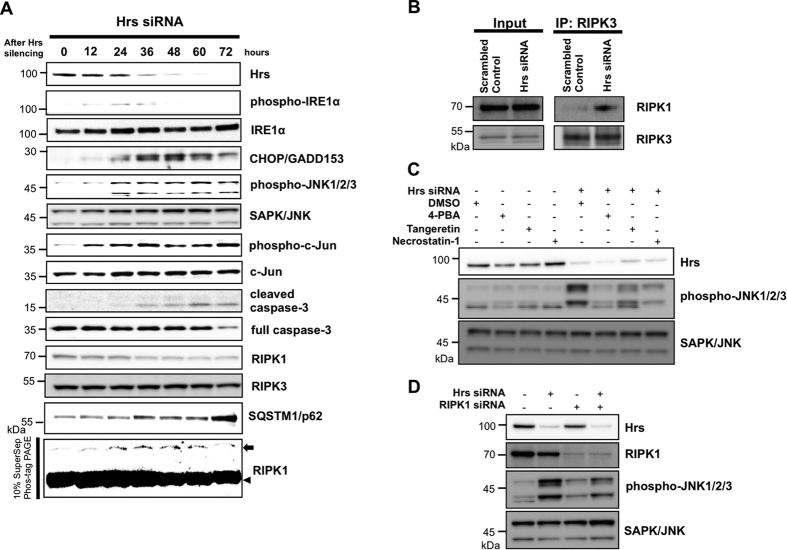
ER stress induction is a prerequisite for the JNK-mediated apoptotosis and necroptosis in PC12 cells. (**A**) Temporal expression profiles of ER stress markers, JNK signaling intermediates, caspase-3 and RIPK1 (a marker for necroptosis) in Hrs-silenced PC12 cells. For electromobility shift-based detection of phosphorylated RIPK1, samples were separated by 10% Phos-tag^®^ gels. The black arrowhead and arrow indicate total and phosphorylated RIPK1, respectively. (**B**) Co-immunoprecipitation revealed a physical interaction between RIPK1 and RIPK3 in Hrs-silenced PC12 cells. (**C**) The expression of phosphorylated JNK1/2/3 in Hrs-depleted cells was markedly suppressed by the ER stress inhibitors (4-PBA and tangeretin) or the RIPK1-specific inhibitor (necrostatin-1). (**D**) JNK1/2/3 phosphorylation in Hrs-depleted PC12 cells was reduced by the genetic ablation of RIPK1.

Although various types of stress can activate the JNK pathway, ER stress can trigger neuronal apoptosis through the activation of the inositol-requiring kinase 1 (IRE1)-apoptosis signal regulating kinase 1 (ASK1)-MKK4-JNK signaling pathway in PD and ALS models^18^. It is known that the inhibition of autophagy potentiates ER stress and subsequent apoptotic and non-apoptotic cell death^19^,^20^. Reciprocally, ER stress can induce autophagy, which results in the degradation of unfolded proteins^21^,^22^. Thus, if autophagic degradation is perturbed by Hrs dysfunction, it could be possible that an excess amount of unfolded, aggregated proteins induces sustained activation of ER stress and upregulation of the downstream JNK signaling cascade, which may ultimately lead to cell death. To evaluate whether the neurotoxicity induced by Hrs silencing was dependent on ER stress activation and subsequent JNK signaling, we monitored the expression profiles of ER stress markers (phospho-IRE1α and C/EBP-homologous protein (CHOP)), JNK signaling intermediates, caspase-3 and the markers of programmed necrosis in Hrs-silenced PC12 cells. In a detailed time course experiment, endogenous Hrs started to decrease by 12 h and totally disappeared after 48 to 60 h (Fig. 5A). Phosphorylated IRE1α, a known ER stress sensor, was already observed after 12 h and reached a maximum level at 24 h. This increase was followed by the prolonged induction of the late ER stress marker CHOP beyond 60 h. Phosphorylation of JNK1/2/3 and c-Jun first became apparent at 12 h and was continually activated to the prolonged time point of 72 h. Consequently, the apoptosis effector caspase-3 began to be activated at approximately 36 h. As we demonstrated by flow cytometric analyses (Fig. 4B), the silencing of Hrs appears to induce both apoptotic and necrotic cell death. Apoptosis has long been believed to be the sole form of programmed cell death, whereas necrosis has been regarded as a passive process without any underlying regulatory mechanisms. However, this notion has recently been challenged, and compelling evidence has emerged that suggests that some types of necrosis are, in fact, programmed cell death; this process has been termed necroptosis^23^. Necroptosis is defined as a distinct form of cell death that is caspase-independent and mediated through formation of the receptor-interacting serine/threonine kinase (RIPK) 1/ RIPK3 complex^24^,^25^,^26^,^27^. Based on these observations, we then attempted to investigate the potential involvement of necroptosis in the Hrs-depleted cells. As expected, after the silencing of Hrs in PC12 cells, a time-dependent increase in phosphorylated RIPK1, a molecular determinant of necroptosis, was observed, as indicated by the electrophoretic mobility shift on the Phos-tag^®^ PAGE gel (Fig. 5A). Furthermore, co-immunoprecipitation revealed that a RIPK1-RIPK3 necrosome complex formation was observed in the Hrs-silenced PC12 cells (Fig. 5B). Moreover, we found that both ER stress inhibitors (4-phenylbutyrate (4-PBA) and tangeretin) and a RIPK1-specific inhibitor (necrostatin-1) successfully reduced phospho-JNK1/2/3, indicating that ER stress induction and RIPK1 activation are likely to be prerequisites for the activation of JNK signaling in Hrs-depleted cells (Fig. 5C). The effect of RIPK1 on downstream JNK activation was also confirmed by the fact that RIPK1 silencing partially relieved the phosphorylation of JNK1/2/3 (Fig. 5D).

### Necroptosis inhibitor as well as pan-caspase inhibitors partially ameliorated neurotoxicity in Hrs-depleted cells

**Figure 6:**
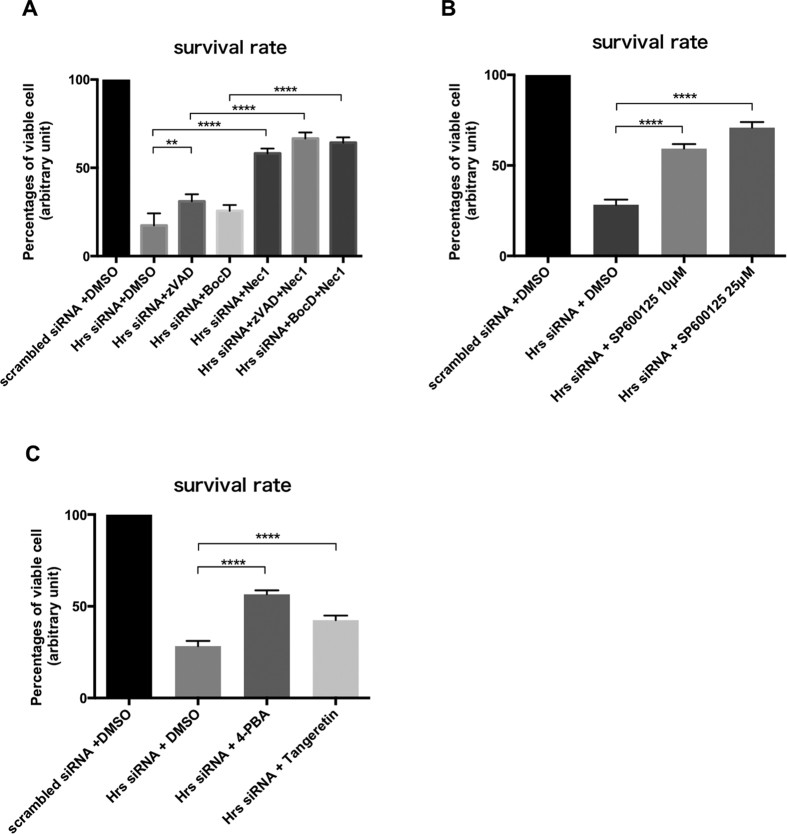
Necroptosis inhibitor as well as pan-caspase inhibitors partially ameliorated neurotoxicity in Hrs-depleted cells. (**A**) Treatment with pan-caspase inhibitors (zVAD-fmk or BocD-fmk) or necrostatin-1 (Nec1) significantly counteracted the neurotoxicity induced by the silencing of Hrs. Values are expressed as means ± standard errors. **p < 0.01, ****p < 0.001 (one-way ANOVA followed by Dunnett’s test; n = 4). (**B**) SP600125 (JNK specific inhibitor) significantly counteracted the neurotoxicity induced by the silencing of Hrs. Values are expressed as means ± standard errors. ****p < 0.001 (one-way ANOVA followed by Dunnett’s test; n = 4). (**C**) ER stress inhibitors (4-PBA and tangeretin) significantly ameliorated the neurotoxicity induced by the silencing of Hrs. Values are expressed as means ± standard errors. ****p < 0.001 (one-way ANOVA followed by Dunnett’s test; n = 4).

Next we tried to rescue the Hrs-mediated neuronal cell death using specific inhibitors for caspases (zVAD-fmk and BocD-fmk), necroptosis (necrostatin-1), JNK (SP600125), and ER stress (4-PBA and tangeretin). As demonstrated by the MTT cell viability assay (Fig. 6A–C), these regents effectively prevented the neuronal cell loss in Hrs-silenced PC12 cells. Note that even with the most effective concentrations tested, the cytoprotective effect of the caspase inhibitors was limited compared to that of necrostatin-1, SP600125, 4-PBA, and tangeretin.

### Hrs-depletion causes the upregulation of ER stress marker, apoptosis and necroptosis signaling molecules in primary hippocampal neurons and mice brain

**Figure 7:**
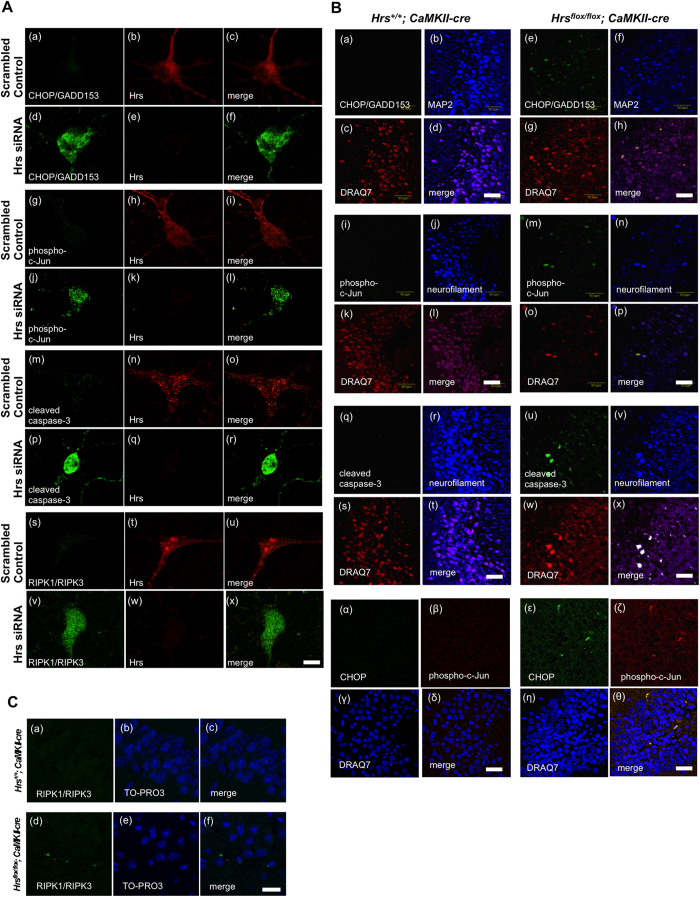
Hrs-depletion causes the upregulation of ER stress marker, apoptotosis and necroptosis signaling molecules in primary hippocampal neurons and mice brain. (**A**) Rat hippocampal primary neurons were immunostained with antibodies against CHOP/GADD153 (a–f), phospho-c-Jun (g–l), and cleaved caspase-3 (m–r) together with anti-Hrs antibody. Molecular interaction between RIPK1 and RIPK3 was observed as a green fluorescent signals using Duolink *In Situ* PLA kit (s-x). Scale bar, 10 μm. (**B**) Brain sections of hippocampal area CA3 from 7-week-old *Hrs*^+/+^*; CaMKIIα-cre* and *Hrs*^*flox/flox*^*; CaMKIIα-cre* mice were immunostained with antibodies against CHOP/GADD153 (a, e, α, ε), phospho-c-Jun (I, m, β, ζ), and cleaved caspase-3 (q, u). MAP2 and neurofilament were used as neuronal markers. Nuclei were counterstained with DRAQ7. Scale bar, 50 μm. (**C**) RIPK1 and RIPK3 complex formation was visualized in the brain sections of hippocampal area CA3 from 7-week-old *Hrs*^+/+^*; CaMKIIα-cre* and *Hrs*^*flox/flox*^*; CaMKIIα-cre* mice using a Duolink *In Situ* PLA kit (a, d). Nuclei were counterstained with TO-PRO3. Scale bar, 20 μm.

To further confirm the results obtained from the Hrs-silenced PC12 cells, we observed the expression of ER stress markers as well as signaling intermediates of apoptosis and necroptosis using rat primary hippocampal neurons and the brain tissues of Hrs-knockout mouse. As shown in the immunocytochemical images, Hrs-silenced primary hippocampal neurons showed the higher expression of CHOP (Fig. 7A, d–f), phospho-c-Jun (Fig. 7A, j–l), and cleaved caspase-3 (Fig. 7A, p–r) compared to mock-treated cells. Likewise, the results of immunostaining of the mouse brain revealed that the *Hrs*^flox/flox^; *CaMKIIα-cre* mice had higher expression of CHOP (Fig. 7B, e–h), phospho-c-Jun (Fig. 7B, m–p), and cleaved caspase-3 (Fig. 7B, u–x) in the CA3 region of the hippocampus compared to the control mice. Of note, the increased expression of CHOP and phospho-c-Jun in the hippocampus of Hrs-knockout mouse was observed in the same cells (Fig. 7B, ε-θ). Moreover, an *in situ* proximity ligation assay (DuoLink^®^) demonstrated that the assembly of RIPK1-RIPK3 complex was detected both in Hrs-silenced primary hippocampal neurons (Fig. 7A, v–x) and the brain of Hrs knockout mice (Fig. 7C, d–f). Collectively, these findings suggest that Hrs-depletion in the nervous tissues induces the upregulation of ER stress marker and eventually leads to both apoptotic and necroptotic cell death.

## Discussion

**Figure 8:**
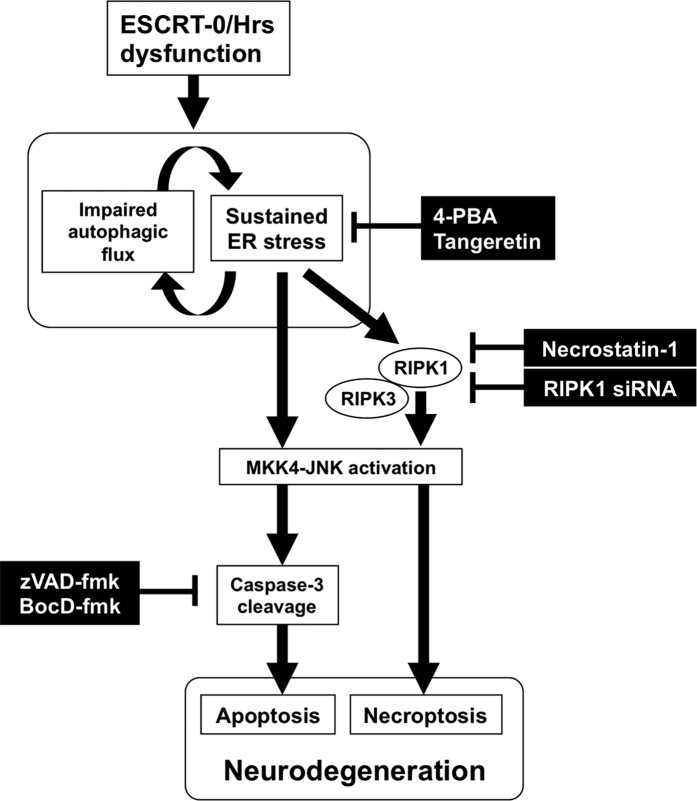
Schematic presentation of neuronal cell death induced by ESCRT-0/Hrs dysfunction. Loss of Hrs leads to a vicious cycle of autophagic impairment and ER stress, which induces the activation of downstream MKK4-JNK signaling and subsequent caspase-dependent apoptosis, which can be attenuated by pan-caspase inhibitors. Alternatively, activation of the ER stress response induces necroptosis through activation of the RIPK1/RIPK3-JNK signaling cascade. This process can be restored by pharmacological or genetic suppression of RIPK1.

In this study, we first demonstrated that the ablation of ESCRT-0/Hrs in the mouse forebrain resulted in marked hippocampal neuronal cell loss, which was proceeded by the accumulation of ubiquitinated proteins, including αS, TDP-43 and huntingtin in addition to p62. Second, we found that RNAi-mediated silencing of Hrs in PC12 cells induced the accumulation of p62 and that autophagic flux was impaired. As a result of Hrs depletion in primary hippocampal neurons and the brain of Hrs-knockout mice, both apoptotic and necrotic cell death occurred, which was mediated through the elevation of ER stress followed by the activation of JNK1/2/3. Finally, we found that RIPK1, a master regulator of necroptosis, was activated by Hrs silencing and that a RIPK1 inhibitor, necrostatin-1, partially prevented the neurotoxicity, as did pan-caspase inhibitors. Cumulatively, these findings suggest that the disruption of Hrs in the mammalian central nervous system compromises autophagic/lysosomal degradation of aggregate-prone proteins, which simultaneously triggers ER stress-mediated apoptotic and necroptotic cell death (Fig. 8). The *Hrs*^*flox/flox*^; *CaMKIIα-cre* mice in this study exhibited more severe neuronal cell loss as well as more profound neuropathological findings than the *Hrs*^*flox/flox*^; *synapsin I-cre* mice we generated previously^24^. In the latter mice, the affected areas were limited to hippocampal area CA3 and the dentate gyrus, whereas the *Hrs*^*flox/flox*^; *CaMKIIα-cre* mice showed even more severe degeneration throughout the hippocampus and cortex. Since an *in situ* hybridization study has shown that Hrs is predominantly expressed in hippocampal neurons in the mouse brain, the severe phenotype of the *Hrs*^*flox/flox*^; *CaMKIIα-cre* mice could be interpreted as a result of near-complete depletion of Hrs in the forebrain, enabled by the distinct spatiotemporal pattern of the expression of Cre recombinase driven by the *CaMKIIα* promoter^25^.

Hrs silencing in the mouse brain and in cultured neurons considerably affected autophagic clearance of ubiquitinated proteinaceous aggregates. Our study suggests an arrest of autophagosome fusion with acidic compartments including late endosomes and lysosomes. This is in line with previous observations that dysfunction of the class E Vps proteins Vps4p/SKD1 in *Saccharomyces cerevisiae* and CeVPS-27 in *Caenorhabditis elegans* as well as of Hrs in HeLa cells impedes the formation of autolysosomes^26^,^27^,^28^. Because ESCRTs are involved in MVB biogenesis but have not been clearly demonstrated to mediate membrane fusion, it remains uncertain how ESCRT-0/Hrs mediates the process of autophagosome fusion. Furthermore, a fundamental issue is that any factors that affect endo-lysosomes could secondarily hamper autophagosome-lysosome fusion or autophagic degradation, making it difficult to identify authentic fusion factors. To address this, it would be interesting to explore functional relationship between ESCRTs and the known molecules required for autophagosome fusion such as Rab7 GTPase, SNARE and HOPS (homotypic fusion and vacuole protein sorting) complex^29^,^30^,^31^. Importantly, we found that depletion of Hrs caused extensive accumulation of the autophagic adaptor molecule p62 as well as of ubiquitinated protein aggregates in neuronal cells. Hrs binds ubiquitinated proteins, and the tagging is required for ESCRT’s selective sorting function, whereas p62-associated ubiquitin and ubiquitination *per se* is often a prerequisite for substrate recognition and determines selective autophagy, raising the possibility that autophagosome execution and ESCRT-mediated protein aggregate removal merge at the same tagging in terms of ubiquitination^32^. Indeed, neurodegeneration in humans is often associated with marked increases in ubiquitinated aggregates in cells, and our data raise the possibility that p62-mediated selective autophagy of ubiquitinated proteins and aggregates requires normal ESCRT function. Accordingly, this notion is supported by previous findings that showed that selective autophagy directly contributes to the autophagic clearance of proteinaceous aggregates that has been linked to several neurodegenerative diseases^33^,^34^. The significance of p62-mediated selective autophagy is further strengthened by the finding that showed that silencing Tsg101 (ESCRT-I) or Vps24 (ESCRT-III) in HeLa cells promoted the formation of TDP-43-positive inclusions that were co-localized with both ubiquitin and p62^35^. In addition to these *in vitro* observations, p62 as well as CHMP2B and Vps4 were found to be co-localized with Lewy bodies in PD^13^,^15^,^36^. Taking all these findings into account, it would appear that the buildup of insoluble, neurodegenerative disease-related protein aggregates in Hrs-silenced neurons could be partially interpreted as the consequence of impaired selective autophagy.

Our observation indicates the functional relevance of ER stress in the neurodegenerative process of ESCRT-defective cells. In response to stress conditions, such as nutrient deprivation and hypoxia, autophagy allows the degradation/recycling of protein waste and cellular components, thereby maintaining intracellular homeostasis. However, once the autophagic machinery is perturbed, ER stress-dependent unfolded protein response (UPR) signaling is activated^19^,^20^. On the other hand, UPR can also kick-start autophagic activity, facilitating the degradation of unfolded proteins to alleviate ER stress^22^. Thus, there might be a yin-yang interrelationship between ER stress and autophagy. Supposedly, the loss of ESCRT-0/Hrs in the mouse brain can induce both chronic failure of autophagy, resulting in hazardous protein aggregate formation and enhanced ER stress, which might synergistically lead to a vicious cycle that leads to neuronal cell death. Another possible scenario is that “ER-phagy,” a process of stress-related ER removal by autophagy-like vesicle formation, might be regulated by ESCRT^37^. Further investigations are required to understand the precise cellular mechanisms that connect autophagy, ER stress and ESCRT function.

The strong activation of JNK due to Hrs silencing found in this study seems to be a key event that facilitates neuronal death. Activation of JNK has been implicated in a number of forms of stress-induced apoptosis^38^. Supporting this notion, murine JNK1/2^−/−^ fibroblasts are resistant to stress-induced apoptosis, and JNK3^−/−^ neurons are insensitive to stimulation-induced apoptosis^39^,^40^. It is of note that, in addition to the JNK-caspase-dependent apoptotic pathway, RIPK1-JNK-dependent necroptosis also appears to be involved in Hrs-silenced PC12 cells. This is interesting since the involvement of necroptosis has increasingly been recognized in the pathogenesis of a wide range of neurological disorders including PD, ALS, stroke and multiple sclerosis^41^,^42^,^43^,^44^. There are different necroptosis initiators for different disease conditions that are followed by specific downstream signaling molecules; however, tumor necrosis factor-α receptor (TNFR)-induced necroptosis is the most extensively studied^45^. In this study, we failed to find up-regulation of TNFR expression (data not shown); as a result, the involvement of TNFR-mediated signaling in Hrs deficiency remains unclear. Given that the neurotoxicity that accompanied JNK phosphorylation in the Hrs-deficient cells was markedly suppressed in the presence of necrostatin-1, JNK may be a candidate intermediary that connects Hrs silencing to necroptotic cell death. Indeed, recent evidence has aroused increasing attention regarding the functional linkage between JNK and the necroptosis pathway. For instance, the RIPK1/RIPK3 kinase cascade regulates mitochondrial oxidative stress through JNK activation^46^,^47^. In other cellular models, mixed lineage kinase domain-like protein (MLKL), a molecule downstream of RIPK3, is required for ROS generation and for the late phase of JNK activation^48^. More recently, TNFR stimulation has been reported to facilitate JNK-mediated necroptosis, possibly through poly(ADP-ribose) polymerase (PARP) 1 activation^49^. Although there have been many reports indicating that necroptosis takes place upon death receptor activation in conditions where apoptosis is blocked, it could be possible that the two pathways are employed by cells in a parallel, complementary fashion to facilitate cellular demise. Indeed, at the level of signaling, several recent studies have shed light on the close interplay between apoptosis and necroptosis. For example, analysis of FADD/RIP3 and FLIP (FLICE inhibitory protein)/RIP3 double knockouts revealed cross-regulation of apoptosis and necrosis^50^.

In summary, we demonstrated that forebrain-specific ablation of ESCRT-0/Hrs induced hippocampal neuronal cell loss, which was accompanied by the accumulation of neurodegenerative disease-related proteins in addition to p62. In particular, our work offers the novel insight that the loss of Hrs in neuronal cells impaired the late stage of autophagic flux as well as induced ubiquitinated protein aggregates and ER stress-mediated apoptotic and necroptotic cell death. In addition, we showed that RIPK1, JNK and caspase inhibitors significantly attenuated cellular damage due to ESCRT-0 dysfunction, suggesting novel therapeutic approaches for the incurable, devastating neurodegenerative disorders. Further investigation of ESCRT-related vesicular trafficking pathways in neuronal cells will pave the way for better understanding of the pathophysiology of these diseases.

## Methods

### Generation of Hrs conditional knockout mice

To generate forebrain-specific Hrs-deficient mice, *Hrs*^*flox/flox*^ mice on a C57BL/6 background were bred with *CaMKIIα-cre* mice on the same background (a generous gift from Dr. Katsuhiko Mikoshiba, Riken BSI, Saitama, Japan), and the progeny were crossed to yield homozygous knockout (*Hrs*^*flox/flox*^; *CaMKIIα-cre*) and control (*Hrs*^+/+^; *CaMKIIα-cre*) mice. The mice were genotyped by PCR with genomic DNA obtained from tail biopsies. All protocols involving animals were approved by Miyagi Cancer Center and the Institutional Animal care and Use Committee; experiments involving animals were performed in accordance with the relevant approved guideline and regulation.

### Histology and immunohistochemistry

The animals were deeply anesthetized and perfused with 4% paraformaldehyde. The brains were removed and postfixed for 2 h in 4% paraformaldehyde before being embedded in paraffin. Antigen retrieval was performed by soaking the brains in citrate buffer for 30 minutes at 90 °C. Endogenous peroxidase activity was suppressed with 0.03% hydrogen peroxide. Immunostaining was performed by the streptavidin-biotin immunoperoxidase method. The following primary antibodies were used: anti-ubiquitin (MBL) and anti-αS (CST). For histological analyses, sections were Nissl stained according to standard protocols.

### Immunofluorescence confocal microscopy

For immunocytochemistry, cells grown on poly-L-lysine-coated coverslips were fixed with 4% paraformaldehyde for 10 min and permeabilized with 0.5% Triton X-100 in PBS for 10 min. After washing step with PBS, cells were incubated in a blocking solution (2% goat serum in PBS) and then incubated with primary antibodies. For immunohistochemistry, mouse brain sections were prepared according to the method previously described^24^. The following primary antibodies were used: anti-p62 (CST), anti-ubiquitin (Santa Cruz), anti-αS (CST), anti-CHOP (Santa Cruz), anti-phospho-neurofilament (Covance), anti-phospho-c-Jun (CST), and anti-cleaved caspase-3 (CST). Positive immunostaining was detected using Alexa 488- and Alexa 647-conjugated secondary antibodies (Molecular Probes). Nuclei were counterstained with DRAQ7 (BioStatus) or TO-PRO3 iodide (Molecular Probes). Fluorescence images were analyzed with an FV300 confocal laser scanning microscope (Olympus).

### Western immunoblot analysis

Western blot was performed according to a standard protocol. For the mobility-shift detection of phosphorylated proteins, the samples were separated using 10% Super Sep Phos-tag^®^ gels (Wako). After a blocking step, the membranes were incubated with the following primary antibodies: anti-Hrs (Abnova), anti-ubiquitin (Santa Cruz), anti- p62 (CST), anti-αS (BD Bioscience), anti-LC3A/B (CST), anti-TDP-43 (Proteintech), anti-huntingtin (CST), anti-HA (Roche), anti-α-tubulin (Santa Cruz), anti-IRE1α (CST), anti-phospho-IRE1α (GeneTex), anti-phospho-MKK4 (CST), anti-JNK (CST), anti-phospho-JNK1/2/3 (CST), anti-c-Jun (CST), anti-phospho-c-Jun (CST), anti-full caspase-3 (Santa Cruz), anti-cleaved caspase-3 (CST), anti-RIPK1 (R&D Systems) and anti-RIPK3 (CST). The primary antibody labeling was followed by the addition of HRP-conjugated secondary antibodies. The bands were visualized with the Luminata Western HRP substrate (Millipore), and the images were captured using the LAS-3000 mini image analyzer (Fujifilm).

### Sequential detergent-based extraction of mouse brains

All the steps of the extraction scheme were carried out at 0-4 °C with ice-cold reagents. Half of a mouse brain was homogenized in 9 volumes of hypotonic lysis buffer (10 mM Tris-HCl (pH 7.4), 0.1 M sucrose, 10 mM NaCl, 1 mM dithiothreitol plus 1x Cømplete protease inhibitor cocktail; Roche). The homogenate was centrifuged at 12,000 × *g* for 30 min. The supernatant was kept as the “buffer-soluble fraction.” The pellet was resuspended in 1% Triton X-100 in PBS, followed by centrifugation at 12,000 × *g* for 30 min. The supernatant was kept as the “detergent-soluble fraction.” The pellet was further resuspended in RIPA buffer, followed by centrifugation at 12,000 × *g* for 30 min. The supernatant was kept as the “detergent-insoluble fraction.” The pellet was resuspended in 8 M urea in 5% SDS, followed by centrifugation at 12,000 × *g* for 30 min. The supernatant was kept as the “urea-soluble fraction.”

### Co-immunoprecipitation

Co-immunoprecipitation was performed according to a previously described method^51^. In brief, cells were washed twice with ice-cold PBS and lysed in ice-cold TNE buffer containing 50 mM Tris-HCl (pH 7.4), 150 mM NaCl, 0.5% NP-40, 1 mM EDTA and 1x protease inhibitor cocktail (Roche). Lysates containing 500 μg total protein were incubated overnight on a carousel at 4 °C with the first antibody, followed by an additional incubation with protein-G-agarose for 2 h. After 4 washes with TNE buffer containing 0.1% NP-40, the protein complexes were eluted with 2x non-reducing Laemmli buffer at 45 °C for 15 min and subsequently analyzed by Western blotting.

### Cell culture and plasmid transfection and RNA interference

PC12 rat pheochromocytoma cells were maintained in DMEM supplemented with L-glutamine and 10% FBS at 37 °C in humidified condition of 5% CO_2_/air. In some experiments, the cells were incubated with medium containing the chemical chaperone 4-PBA (TCI), the UPR activator tangeretin (TCI), pan-caspase inhibitors (zVAD-fmk from MBL and BocD-fmk from Abcam), an allosteric inhibitor of RIPK1 (necrostatin-1; Sigma), and JNK inhibitor (SP600125; LCL). 3 × FLAG-tagged human wt and Q343R mutant TDP-43 expression vectors were kindly gifted from Dr. Koji Yamanaka (Department of Neuroscience and Pathobiology, Nagoya University, Japan)^52^. Plasmids were introduced into the cells using the FuGENE 6 (Roche) according to the manufacturer’s protocol. For RNAi-mediated gene silencing, the cells were transfected with target-specific or control scrambled siRNA using Lipofectamine RNAiMAX (Life Technologies). The following small interfering RNAs (siRNAs) were used: rat Hrs siRNA#1, 5′-CCAUCAAGAAGAAGGUCAAUGAUAA-3′; rat Hrs siRNA#2, 5′-UUAUCAUUGACCUUCUUCUUGAUGG-3′; and rat RIPK1 siRNA, 5′-GCGCUGAGUACAAUGAGGCUCUCUU-3′. Scrambled control siRNA (sc-37007) was purchased from Santa Cruz Biotechnology.

### Culture of primary rat hippocampal neuron

Rat hippocampal neurons obtained from the late embryonic stage (E19) were prepared according to the previous method with slight modification^53^. Dissociated hippocampal neurons were electroporated with rat Hrs siRNA using the NEPA21^®^ electroporator (NEPA Gene). After transfection, cells were plated at a density of 0.5 × 10^6^ cells on a poly-L-lysine-coated Lab-Tek^®^ II chamber slide, and cultured in Neurobasal A medium supplemented with 2% B27, 25 mM glutamate, 18 mM glucose and 0.5 mM L-glutamine. Forty-eight hours after initiation of culture, cells were subjected to immunocytochemical analysis.

### MTT cell viability assay

Cell survival rates were evaluated using the colorimetric MTT assay as described previously^54^.

### Flow cytometric measurement of apoptotic and necrotic cells

An FITC Annexin V Apoptosis Detection kit (BioLegend) was used to detect apoptosis and necrosis. Briefly, the cells were suspended in 100 μl of Annexin V binding buffer within the range of 1 × 10^7^ cells/ml and mixed with 5 μl of FITC-conjugated Annexin V and 7-AAD. After incubation at room temperature for 15 min in the dark, 400 μl of Annexin V binding buffer was added to the mixtures. The stained cells were then analyzed using a BD FACSCanto^TM^ II flow cytometer (BD Bioscience).

### Monitoring autophagic flux

PC12 cells that were treated with control or Hrs target-specific siRNA were incubated for 72 h before the addition of the Premo^®^ autophagy tandem sensor RFP-GFP-LC3B (Life Technologies). After 16 hours of incubation with BacMam RFP-GFP-LC3B particles, the cells were visualized using confocal laser scanning microscopy. Chloroquine (Sigma), an inhibitor of lysosomal acidification, was used to prevent the later step of autophagic degradation.

### PathScan intracellular signaling arrays

The slide-based antibody array (PathScan^®^ Intracellular Signaling Array kits; CST) for simultaneous detection of 18 signaling molecules, including stress-activated protein kinases were analyzed according to the manufacturer’s protocol and scanned using the Omega Lum^®^ G image analysis system (Aplegen).

### *In situ* proximity ligation assay

Rat primary hippocampal neurons or paraffin-embedded mouse brain sections were fixed and incubated overnight with anti-RIPK1 and anti-RIPK3 antibodies. To visualize the RIPK1-RIPK3 complex *in situ*, cells were further incubated with Duolink^®^ *In Situ* PLA reagents (Sigma) according to the manufacturer’s protocol. Green fluorescent DuoLink signals were observed under a confocal microscope.

### Statistical analysis

Pooled data from at least 4 independent experiments were statistically analyzed using GraphPad Prism version 6 (GraphPad Software). For comparisons between two groups, Student’s *t*-test was adopted. In the case of multiple comparisons, one-way analysis of variance (ANOVA) with post hoc Dunnett’s test was used. The data were reported as means ± standard errors.

## Additional Information

**How to cite this article**: Oshima, R. *et al.* ESCRT-0 dysfunction compromises autophagic degradation of protein aggregates and facilitates ER stress-mediated neurodegeneration via apoptotic and necroptotic pathways. *Sci. Rep.* **6**, 24997; doi: 10.1038/srep24997 (2016).
